# Enhancing critical thinking skills and self-directed learning among critical care nursing students: a quasi-experimental study on the impact of the modified KWHLAQ strategy

**DOI:** 10.1186/s12912-026-05254-y

**Published:** 2026-09-28

**Authors:** Yasmeen Mohamed Mohamed Shehata, Nourhan Essam Hendawi, Mohamed H. R. Atta, Mohamed Ahmed Aly, Amany Saeed Abdelaziz Nagy, Ahmed Abdelhafeez Abdelmonseif Younis

**Affiliations:** 1 Nursing Education Department, Faculty of Nursing, Alexandria University, Alexandria, Egypt; 2 Nursing Department, College of Applied Medical Sciences, Prince Sattam Bin Abdulaziz University, Wadi Addawasir, Saudi Arabia; 3 Psychiatric and Mental Health Nursing Department, Faculty of Nursing, Alexandria University, Alexandria, Egypt; 4 Faculty of Nursing, Nursing Department, College of Applied Medical Sciences, Helwan University, Prince Sattam Bin Abdulaziz University, Wadi Addawasir, Saudi Arabia; 5 Nursing Education, Faculty of Nursing, Alexandria University, Alexandria, Egypt; 6 Emergency and Critical Care Nursing, Faculty of Nursing, Alexandria University, Alexandria, Egypt; 7 Nursing Department, College of Applied Medical Sciences, Prince Sattam Bin Abdulaziz University, 18734 Wadi Addawasir, Saudi Arabia

**Keywords:** Problem-based learning, Clinical decision making, Nursing, Intensive care, Critical thinking, Nursing education, Quasi-experimental study, Self-directed learning

## Abstract

**Objective:**

To evaluate the effect of the modified Know–Want–Learn–How–Apply–Questions (KWHLAQ) strategy on critical thinking and self-directed learning among third-semester undergraduate nursing students enrolled in a critical care nursing course.

**Methods:**

A quasi-experimental, nonequivalent control group pretest–posttest design was employed. One hundred undergraduate nursing students (*n* = 50 per group) enrolled in the Critical and Emergency Nursing Department, Faculty of Nursing, Alexandria University, participated during the 2025 academic session. The study group received a 12-week structured intervention based on the KWHLAQ framework; the control group received standard lecture-based instruction. Both groups completed the Critical Thinking Questionnaire (CThQ; α = 0.80) and the Self-Directed Learning Instrument (SDLI; α = 0.80) before and after the intervention.

**Results:**

After covariate adjustment, the study group demonstrated significantly higher post-intervention critical thinking scores than the control group (*p* < 0.05; Cohen’s d = 0.67, moderate-to-large effect). The study group improved significantly in overall critical thinking (mean difference = 5.66; *p* < 0.01) and in the evaluating subscale (mean difference = 4.50; *p* < 0.001; Cohen’s d = 2.10, whereas the control group’s scores declined. Self-directed learning improved substantially in the study group (mean difference = 23.06; *p* < 0.001; Cohen’s d = 2.45), with significant gains across all four SDLI subscales.

**Conclusion:**

Integration of this structured, low-cost, and replicable pedagogical framework into nursing curricula may promote active learning, reflective reasoning, and clinical decision-making competencies essential for high-acuity practice.

## Introduction

Nursing students are moving beyond basic clinical skills to acquire more sophisticated competencies as patients’ health conditions become increasingly complex and healthcare systems continue to evolve. Higher-order cognitive and professional skills, particularly critical thinking and self-directed learning, are essential for preparing students for professional practice in critical care settings where clinical situations are dynamic and patient outcomes often depend on timely and evidence-based decisions [1, 2].

In critical care nursing education, students are frequently exposed to high-acuity clinical scenarios involving patients experiencing life-threatening conditions, rapid physiological deterioration or instability, and complex treatment requirements. These learning situations involve ongoing assessments, clinical data interpretation, and prioritization of numerous interventions in real time under pressure. Thus, in critical care courses, teaching-learning methods that enhance students’ ability to analyze information and respond correctly are of great importance [3, 4].

Critical thinking (CT) is a cognitive process that enables nursing students to analyze information systematically, evaluate evidence, and make reasoned judgments in clinical situations. These skills help students distinguish between facts, assumptions, and opinions while applying logical reasoning and problem-solving strategies to patient care. In critical care environments, where patients may experience rapid physiological changes and require immediate interventions, CT enables nursing students to adapt quickly, prioritize care, and respond effectively to evolving clinical situations, thereby improving patient safety and quality of care [5–7].

Critical thinking encompasses not only cognitive skills but also intellectual dispositions such as open-mindedness, reflection, perseverance, intellectual honesty, and contextual awareness, all of which contribute to sound clinical judgment. Evidence-based decision-making is closely linked to CT because it requires students to integrate research findings with clinical knowledge and experience. Strategies such as critical analysis and Socratic questioning encourage deeper inquiry, allowing students to evaluate multiple perspectives, assess the credibility of information sources, and justify clinical decisions. Furthermore, CT supports students’ participation in interprofessional collaboration, patient advocacy, and quality improvement initiatives [8, 9].

Although clinical reasoning is recognized as a context-specific process through which nurses collect, interpret, and respond to patient information, critical thinking remains one of its fundamental cognitive foundations. Critical thinking provides the analytical, evaluative, and reflective abilities necessary for effective clinical reasoning and clinical judgment. Previous nursing education research has demonstrated that strong critical thinking skills contribute significantly to sound clinical decision-making and reasoning performance. Therefore, critical thinking was selected as a primary outcome in the present study because it represents a core transferable competency that underpins effective clinical reasoning in critical care practice [10–12].

Self-directed learning (SDL) is another essential competency that complements critical thinking. SDL is a process in which learners take responsibility for identifying their learning needs, setting goals, locating learning resources, selecting appropriate learning strategies, and evaluating learning outcomes. Through SDL, students actively manage their own learning experiences using cognitive and metacognitive strategies that enhance engagement and academic achievement [13, 14].

Self-directed learning offers several educational benefits, including the development of research and analytical skills, increased learner autonomy, greater motivation, and lifelong learning habits. Engagement in SDL is influenced by both external factors, such as learning environments and resource availability, and internal factors, including learning preferences, self-efficacy, and readiness to assume responsibility for learning [15, 16]. Promoting SDL among nursing students is particularly important because it enhances professional competence, confidence, communication abilities, and adaptability in changing healthcare environments [17].

Contemporary nursing education increasingly emphasizes learner-centered approaches that encourage reflection, goal setting, active participation, and knowledge construction. One widely recognized strategy is the Modified Know–Want–Learn (KWL) framework, originally developed by Ogle (1986). The KWL strategy is a structured pedagogical approach designed to foster active learning, self-regulation, and reflective thinking. Students begin by identifying what they already know (K), determining what they want to learn (W), and reflecting on what they have learned (L) throughout the learning process [18–20].

An extended version of this framework, known as KWHLAQ, incorporates three additional components: How can I learn more (H), how I will apply the knowledge (A), and questions that emerge during learning (Q). By guiding learners through these six interconnected stages, KWHLAQ promotes active engagement, reflective inquiry, knowledge application, and self-directed learning [21, 22].

The KWHLAQ strategy is particularly relevant in critical care nursing education because it supports learning in environments characterized by complex patient conditions, rapidly changing clinical situations, and demanding decision-making requirements under pressure. In such settings, students must continuously interpret large volumes of patient data, recognize rapid changes in physiological status, prioritize interventions, and justify their decisions using evidence-based knowledge. The structured sequence of KWHLAQ facilitates these processes by guiding learners through knowledge activation (K), identification of learning needs (W), information seeking (H), reflection (L), application of interventions (A), and continuous generation of new questions to adapt to evolving clinical scenarios (Q). This structured cognitive pathway promotes active engagement, reflective thinking, evidence-based decision-making, and adaptability, which are essential competencies in critical care nursing practice [23, 24].

Despite the documented benefits of KWL-based strategies in promoting active learning, reflective thinking, and learner engagement across educational settings, evidence regarding the effectiveness of the full KWHLAQ framework in nursing education remains limited. In particular, little is known about whether the additional components of information seeking (H), knowledge application (A), and reflective questioning (Q) contribute to enhancing critical thinking and self-directed learning among critical care nursing students. Given the cognitive and educational demands of critical care nursing, identifying effective learner-centered strategies that strengthen these competencies is essential. Therefore, this study aimed to examine the impact of the modified KWHLAQ strategy on critical thinking and self-directed learning among critical care nursing students.

### Theoretical framework

Methodologically, the research followed the (post)positivist paradigm because the study’s quasi-experimental design and hypothesis-testing nature, as well as the use of standardized data analysis and statistical inference, align better with this paradigm than with any other. The intervention was based on the constructivist learning theory rather than on the research paradigm. Particularly, the constructivist theory by Vygotsky provides the rationale for the intervention and its design because it views learning as an active process of constructing knowledge and emphasizes the role of interactions in learning. A key component of this structure is the idea of the Zone of Proximal Development (ZPD), the difference between what a learner can do alone and what they can do with the support of an expert. This study used scaffolding as an operating definition of the structured progression of the KWHLAQ pre-session activation of prior knowledge (K and W components) and facilitated students’ choice of information-seeking strategies for critical care scenarios based on evidence. Throughout the session, the scaffold was systematically removed, and the students were able to independently complete the L, A, and Q sections, reinforcing their learning and creating new clinical questions. This graduated release of responsibility reflects Vygotsky’s scaffolding theory and was used throughout all 12 weeks [25, 26].

With the growing need for nurses who are able to think critically, adapt to the ever-changing clinical setting, and continue learning throughout life, innovative educational strategies have become essential and are no longer optional. The KWHLAQ strategy, based on the constructivist principles of active construction of knowledge, guided learning, and progressive learner autonomy, is one of the strategies that is able to meet these expectations. The strategy provides students with opportunities to question, reflect, apply, and extend their learning beyond the classroom, resulting in a close match with the competencies needed for today’s nursing practice. In this context, the following study was conducted to assess the effectiveness of the KWHLAQ strategy in enhancing the ability of undergraduate nursing students to think critically and learn independently, in order to produce competent and reflective future nurses.

## Methods

### Study design

A quasi-experimental, nonequivalent control group pretest–posttest design was used to evaluate the effect of the modified KWHLAQ strategy on nursing students’ critical thinking and self-directed learning. This study is reported in accordance with the Transparent Reporting of Evaluations with Nonrandomized Designs (TREND) statement.

### Setting

The study was conducted at the Critical and Emergency Nursing Department, Faculty of Nursing, Alexandria University, Egypt. Data were collected during the summer academic session (June–August 2025), which represents the third semester of the academic calendar for the department. Both groups were taught in designated lecture halls in the department’s predetermined timetable, in a controlled academic environment.

### Participants and sampling

A convenient sample of 100 undergraduate nursing students who were enrolled in the Critical and Emergency Nursing course during the third semester of the 2025 academic session was recruited. The criteria for eligibility were that they were enrolled in the critical and emergency nursing course, had attended 90% or more of the scheduled classes, and were willing to participate. Students who had prior experience with KWL or KWHLAQ strategies or who could not commit to the entire 12-week program were excluded.

Sample size was calculated a priori using G*Power 3.1 software. A repeated measures ANCOVA with two groups and two measurement time points, with a moderate effect size (Cohen’s f = 0.25), alpha = 0.05, and power = 0.80 required a minimum sample of 82 participants. One hundred students were recruited to account for possible attrition and to increase statistical power. Students were allocated equally into a study group (*n* = 50) and a control group (*n* = 50). Allocation was an attempt to minimize bias, although the design was quasi-experimental because randomization was not possible, as students were assigned to pre-existing class sections. To reduce group contamination, the sessions were conducted on different class days.

In particular, the study and control groups were significantly different in several demographic variables, notably age (study group: predominantly 18–23 years; control group: predominantly older than 23 years), sex distribution, GPA, and academic year. These imbalances reflect the nature of convenience sampling from non-randomized class sections and are dealt with analytically through ANCOVA covariate adjustment (see Data Analysis). These demographic differences are also noted as a major limitation.

### Study instruments

Both instruments were originally developed in English and administered to participants in English, consistent with the language of instruction at the Faculty of Nursing, Alexandria University. The research team made minor linguistic adaptations to make the instruments culturally appropriate for the context of Egypt without formal forward–backward translation, as the instruments were used in the original English form.

Demographic data (age, sex, GPA, and academic year) were collected alongside the CThQ at pre-intervention using a structured demographic questionnaire developed by the research team.

1. Critical Thinking Questionnaire (CThQ). The CThQ was developed by Kobylarek, Blaszczynski, and Slosarz (2022) and consists of a 25-item measure assessing six domains from Bloom’s revised taxonomy: analyzing, evaluating, creating, remembering, understanding, and applying. Items are scored on a five-point Likert scale (1 = strongly disagree to 5 = strongly agree); four items [4, 9, 19, 22] are reverse scored. Items written in a Likert format were treated as continuous data for subscale and total sum score computation, which is a common practice in measurement research in the educational and psychological fields when the unit of analysis is composite scores and scales have 5 or more ordered response categories. Critical thinking total score ranges from 25 to 125, categorized as low (25–58), moderate (59–92), and high (93–125). Original instrument internal consistency was reported as α = 0.87; pilot testing for the current study yielded α = 0.80.
2. Self-Directed Learning Instrument (SDLI). The SDLI, developed by Cheng et al. (2010), contains 20 items in four subscales: learning motivation, planning and implementation, self-monitoring, and interpersonal communication. Items are rated on a five-point Likert scale (1 = strongly disagree to 5 = strongly agree). As with the CThQ, subscale and total sum scores were computed by summing ordinal Likert responses, consistent with established psychometric practice for validated composite scales. Total scores range from 20 to 100, with low (20–46), moderate (47–73), and high (74–100) categories. The instrument demonstrated good reliability (α = 0.91); the current study confirmed strong internal consistency (α = 0.80). Both instruments were reviewed by a panel of content experts for face and content validity.

### Ethical considerations

Ethical approval was obtained from the Research Ethics Committee of Alexandria University Faculty of Nursing (Approval No. AU-20-9-380). All study procedures were conducted in accordance with the ethical principles of the Declaration of Helsinki. Detailed information about the purpose, procedures, potential risks, and possible benefits of the study was given to all participants. All participants volunteered and gave their written informed consent before the data were collected. Students were explicitly advised that they could withdraw at any time without academic penalty. To maintain confidentiality and anonymity, personal identifiers were replaced with identification numbers. Survey materials were held in locked cabinets and digital files were password-protected, with access restricted to the research team.

### Face validity assessment

A face validity assessment was conducted with 10 students (10% of the required sample) to evaluate the clarity, feasibility, and cultural appropriateness of the research instruments and intervention procedures. This process was not intended as a formal pilot study but rather as an expert and user review to confirm instrument readability and procedural suitability before full data collection. Feedback indicated that both tools were clear and appropriate. Based on participant feedback, minor wording adjustments were made. These 10 students were not included in the main study sample; the final analytical sample comprised 100 independent participants.

### Intervention: the modified KWHLAQ strategy

The intervention was conducted over a structured 12-week period within the Critical and Emergency Nursing course, applying the KWHLAQ framework to high-acuity critical care content areas including hemodynamic monitoring, mechanical ventilation, management of sepsis, and post-operative critical care nursing. The weekly theoretical sessions were organized so that they would reflect the clinical reasoning that nurses have to perform in a critical care context, in which they need to quickly recall previous knowledge, recognize gaps in knowledge, look for evidence, apply evidence to patient cases, and pose new questions, all of which are represented in the six KWHLAQ components. Sessions were conducted using the following structured six-component chart:

- K — What I already know.
- W — What I want to know.
- H — How I can learn more (information-seeking strategies).
- L — What I learned.
- A — How I will apply the knowledge in clinical practice.
- Q — What further questions do I have.

Before each session, students completed the K, W, and H sections, activating prior knowledge, identifying learning needs, and planning information-seeking strategies. This pre-session phase served as scaffolding as per Vygotsky’s ZPD, by having the instructor give guiding prompts about the critical care topic of the session (e.g., “What do you already know about vasopressor management?”) to help connect existing knowledge to the new content. L, A and Q were completed at the end of each session to reinforce learning, identify clinical applications and generate new questions. The A component was designed to specifically link theory with realistic critical care nursing scenarios, with students having to explain how knowledge they have learnt would impact their clinical decision-making and nursing care. This application-to-practice focus was linked to the critical care context and facilitated the acquisition of critical thinking skills that are domain-specific (analyzing, evaluating, creating, remembering, understanding, and applying) as measured by the CThQ. The lead researcher facilitated discussion, encouraged evidence-based reasoning, addressed misconceptions, and provided formative feedback. Intervention fidelity was maintained over the 12 weeks with a standardized implementation guide and session checklist. As an example, in the sepsis management session, students recalled previous knowledge of sepsis pathophysiology using the K component, recognized the gaps in the evidence, including thresholds for fluid resuscitation, using the W component, and planned for evidence-based information-seeking strategies using the H component; and by the end of the session, they used the A component to describe how they would implement the Surviving Sepsis Campaign guidelines in a deteriorating patient scenario while the Q component would elicit new clinical questions, such as the timing of initiating vasopressors.

The control group received standard instruction consisting of faculty-led lectures and discussion sessions. To establish content equivalence, both groups covered identical course material over the same instructional time.

### Data collection procedures

Data collection took place across 12 weeks from June 1 to August 17, 2025, corresponding to the third semester of the 2025 academic session. Pre-intervention assessments (CThQ and SDLI) were administered one day before the first session. All questionnaires were completed individually under research supervision to ensure completeness and to clarify any ambiguities. Post-intervention assessments for both groups were conducted immediately after the final session of the 12 weeks. Data were checked for completeness, coded, and prepared for analysis.

### Data analysis

Data were analyzed using IBM SPSS Statistics, Version 27. Categorical variables were summarized as frequencies and percentages; continuous variables were expressed as means and standard deviations. Likert-scale items from both the CThQ and SDLI were summed to produce subscale and total composite scores, which were subsequently treated as continuous variables in all inferential analyses. This approach is consistent with established conventions in psychometric and nursing education research, where validated instruments with five-point response scales and multi-item composites are routinely analyzed using parametric methods. Normality of continuous variables was assessed using the Kolmogorov–Smirnov test.

Between-group comparisons of categorical demographic variables were conducted using chi-squared or Fisher’s exact tests, as appropriate. Because the two groups differed significantly on several demographic variables (age, sex, GPA, and academic year), analysis of covariance (ANCOVA) was used as the primary analytical approach, with post-intervention scores as dependent variables, group allocation as the independent variable, and pre-intervention scores, age, sex, GPA, and academic year as covariates. This approach statistically controlled pre-existing group differences and pre-intervention score disparities. Paired-samples t-tests were used as supplementary analyses to examine within-group change from pre- to post-intervention. Pearson correlation coefficients were calculated to examine the relationship between critical thinking and self-directed learning. Effect sizes were estimated using Cohen’s d (small: 0.2; medium: 0.5; large: 0.8). Before each ANCOVA, the homogeneity of regression slopes assumption was tested via group × covariate interaction terms; no significant violations were detected. The CThQ provides six subscale scores (remembering, understanding, applying, analyzing, evaluating, and creating), as well as an overall total score. Subscale-level results should be interpreted with caution given the potential for multiple comparisons to increase the likelihood of Type I error; a Bonferroni correction or a conservative cut-off for significance should be used when interpreting subscale scores. All tests were two-tailed; statistical significance was set at *p* < 0.05.

## Results

All outcomes reflect self-reported scale scores; behavioral change was not directly observed.

### Demographic characteristics

Table 1 presents demographic characteristics of both groups. Significant between-group differences were found in age (*p* < 0.001), sex (*p* = 0.013), and GPA (*p* = 0.034), confirming baseline non-equivalence. The study group was predominantly aged 18–23 (96%), while the control group was predominantly older than 23 years (80%). Females comprised 74% of the study group versus 48% of the control group. Most study group participants held a GPA of 3–4 (74%), compared with 50% in the control group. All outcome comparisons were conducted using ANCOVA with demographic variables and baseline scores as covariates.

**Table 1:** Demographic characteristics of study participants (*n* = 100)

Variable	Category	Study Group *N* (%)	Control Group *N* (%)	Test statistic	*p*-value
Age (years)	18–20	14 (28%)	0 (0%)	χ²= 62.4	< 0.001
	21–23	34 (68%)	10 (20%)		
	More than 23	2 (4%)	40 (80%)		
Sex	Male	13 (26%)	26 (52%)	χ²= 6.26	0.013
	Female	37 (74%)	24 (48%)		
GPA	< 2.0	0 (0%)	2 (4%)	χ²= 6.75	0.034
	2.0 – < 3.0	13 (26%)	23 (46%)		
	3.0–4.0	37 (74%)	25 (50%)		

GPA = Grade Point Average. Statistical tests: chi-squared (χ²)

### Critical thinking outcomes

Table 2 presents pre- and post-intervention critical thinking scores and within-group mean changes by group. The study group showed a significant improvement in overall critical thinking from pre- to post-intervention (96.20 ± 7.03 to 101.90 ± 8.69; mean change = 5.70; t = 3.69, *p* = 0.01; Cohen’s d = 0.67). In contrast, the control group showed a significant decline in overall critical thinking scores (101.59 ± 9.94 to 99.35 ± 9.00; mean change = − 2.24; t = 2.33, *p* = 0.05). Among the critical thinking subscales, the largest improvement in the study group was observed in evaluating (mean change = 4.50; t = 15.07, *p* = 0.001; Cohen’s d = 2.10), although this large effect should be interpreted cautiously due to the substantial baseline deficit in the study group. The study group also showed improvement in analyzing (mean change = 0.92; t = 2.23, *p* = 0.05), while changes in creating, remembering, understanding, and applying were small and not statistically significant. In the control group, scores declined across most subscales, with statistically significant or borderline changes observed in critical thinking total score, remembering, and applying.

**Table 2:** Pre- and post-intervention critical thinking scores and mean changes (Post–Pre) by Group (*n* = 50 per group)

Variable	Group	Pre-intervention M ± SD	Post-intervention M ± SD	Mean Diff. (Post–Pre)	Within-group t (*p*)	Cohen’s d
Critical Thinking	Control	101.59 ± 9.94	99.35 ± 9.00	−2.24	2.33 (0.05)	—
	Study	96.20 ± 7.03	101.90 ± 8.69	+ 5.70	3.69 (0.01)	0.67*
Analyzing	Control	18.29 ± 2.57	17.82 ± 2.38	−0.47	1.89 (0.06)	—
	Study	18.32 ± 2.27	19.24 ± 2.12	+ 0.92	2.23 (0.05)	0.42
Evaluating†	Control	16.62 ± 1.91	16.27 ± 1.96	−0.35	1.82 (0.07)	—
	Study	12.06 ± 1.42	16.56 ± 1.73	+ 4.50	15.07 (0.001)	2.10**†**
Creating	Control	23.92 ± 2.57	23.51 ± 2.39	−0.41	1.66 (0.09)	—
	Study	23.20 ± 2.52	23.36 ± 1.98	+ 0.16	0.37 (0.71)	0.09
Remembering	Control	11.23 ± 1.66	10.91 ± 1.55	−0.32	2.02 (0.05)	—
	Study	11.34 ± 1.59	11.38 ± 1.47	+ 0.04	0.13 (0.89)	0.06
Understanding	Control	14.92 ± 2.21	14.68 ± 1.86	−0.23	1.11 (0.27)	—
	Study	14.80 ± 2.24	14.82 ± 1.81	+ 0.02	0.05 (0.96)	0.01
Applying	Control	16.60 ± 1.80	16.15 ± 1.85	−0.45	2.39 (0.05)	—
	Study	16.26 ± 1.52	16.72 ± 1.79	+ 0.46	1.41 (0.17)	0.15

M = mean; SD = standard deviation; Diff. = difference. **†**Cohen’s d values represent within-study-group pre–post standardized mean changes for the intervention group. Note. Mean difference was calculated as post-intervention score minus pre-intervention score. Positive values indicate an increase from baseline, whereas negative values indicate a decrease. Within-group t-values are presented as absolute values; the direction of change is shown by the sign of the mean difference. Positive values indicate post-test improvement over pre-test

### Self-directed learning outcomes

Table 3 shows SDL score changes. Before the intervention, the control group had significantly higher total SDL scores than the study group (63.96 ± 9.56 vs. 53.44 ± 11.54; t = − 8.21, *p* < 0.001), indicating meaningful baseline non-equivalence. After covariate adjustment for pre-intervention SDL scores and demographic variables, the study group reported a significant improvement in total SDL (mean difference = 23.06; *p* < 0.001; Cohen’s d = 2.45) and outperformed the control group at post-intervention assessment (*p* < 0.001). The control group showed no significant overall SDL change (mean difference = − 2.92; *p* = 0.143). Significant gains in the study group were observed across all four SDLI subscales: learning motivation (*p* < 0.001), planning and implementation (*p* < 0.001), self-monitoring (*p* < 0.001), and interpersonal communication (*p* < 0.01). The control group reported a significant decline in planning and implementation (*p* < 0.001), a borderline increase in learning motivation (*p* = 0.053), a significant increase in self-monitoring (*p* = 0.05), and a nonsignificant change in interpersonal communication.

**Table 3:** Pre- and post-intervention self-directed learning scores and mean changes (Post–Pre) by Group (*n* = 50 per group)

Variable	Group	Pre-intervention M ± SD	Post-intervention M ± SD	Mean Diff. (Post–Pre)	Within-group t (*p*)	Cohen’s d
Self-directed learning	Control	63.96 ± 9.56	61.04 ± 11.50	−2.92	1.49 (0.143)	—
	Study	53.44 ± 11.54	76.50 ± 10.16	+ 23.06	9.11 (0.001)	2.45*
Learning motivation	Control	14.18 ± 5.55	16.24 ± 4.99	+ 2.06	1.98 (0.053)	—
	Study	13.60 ± 5.51	18.64 ± 3.88	+ 5.04	6.48 (0.001)	1.10
Planning & implementation	Control	27.76 ± 3.75	20.92 ± 6.32	−6.84	7.46 (0.001)	—
	Study	18.24 ± 4.63	26.90 ± 5.74	+ 8.66	10.32 (0.001)	1.78
Self-monitoring	Control	9.96 ± 3.59	11.68 ± 4.52	+ 1.72	2.58 (0.05)	—
	Study	9.58 ± 4.18	15.54 ± 3.79	+ 5.96	6.97 (0.001)	1.35
Interpersonal communication	Control	12.06 ± 4.63	12.20 ± 4.48	+ 0.14	0.20 (0.842)	—
	Study	12.02 ± 4.46	15.42 ± 4.49	+ 3.40	3.71 (0.01)	0.82

M = mean; SD = standard deviation; Diff. = difference. Mean difference was calculated as post-intervention score minus pre-intervention score. Positive values indicate an increase from baseline, whereas negative values indicate a decrease. Within-group t-values are presented as absolute values; the direction of change is shown by the sign of the mean difference. Positive values indicate post-test improvement over pre-test

### Effect size summary

Table 4 summarizes within-study-group Cohen’s d effect sizes for pre–post changes in the intervention group. Overall critical thinking showed a moderate-to-large effect (d = 0.67). The evaluating subscale showed an apparent very large effect (d = 2.10) that is substantially attributable to the large baseline deficit; this should be interpreted with caution. Creating (d = 0.09), remembering (d = 0.06), understanding (d = 0.01), and applying (d = 0.15) showed negligible to small effects, suggesting that the 12-week KWHLAQ intervention did not extend to higher-order synthesis and knowledge-recall domains within the study period. SDL showed a very large overall effect (d = 2.45), with large-to-very-large effects across all subscales, reflecting the intervention’s strongest impact on self-directed learning scale scores.

**Table 4:** Within-study-group effect sizes (Cohen’s d) for pre–post changes in study outcomes after the modified KWHLAQ strategy – study group only

Variable	Cohen’s d	Interpretation
Overall Critical Thinking	0.67	Moderate-to-large
Analyzing	0.42	Moderate
Evaluating†	2.10**†**	Very large (see note)
Creating	0.09	Negligible
Remembering	0.06	Negligible
Understanding	0.01	Negligible
Applying	0.15	Small
Self-Directed Learning (total)*	2.45*	Very large (see note)
Learning motivation	1.10	Large
Planning and implementation	1.78	Very large
Self-monitoring	1.35	Large
Interpersonal communication	0.82	Large

†Evaluating d = 2.10 reflects a large pre-existing baseline deficit in the study group; interpret with caution. *SDL d = 2.45 reflects change from a significantly lower baseline (see Table 3). Cohen’s benchmarks: small = 0.2; medium = 0.5; large = 0.8. Cohen’s d values indicate pre–post changes within the intervention group only, not adjusted between-group effects. Control group data are not shown in this table because Cohen’s d was computed only for the intervention (study) group to summarize within-group pre–post change after the KWHLAQ intervention; pre–post values for the control group are presented in Tables 2 and 3

**Fig. 1:**
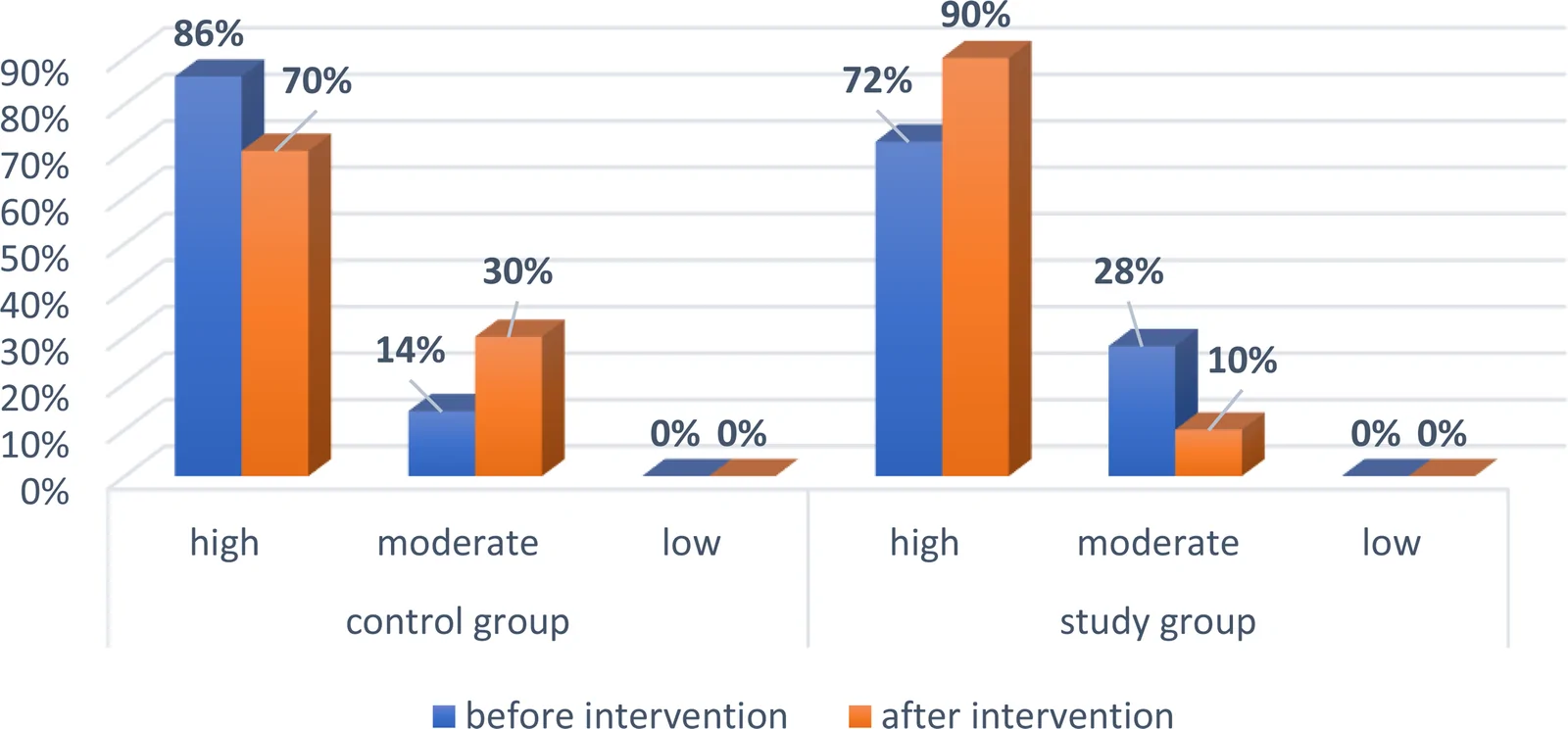
Levels of critical thinking before and after intervention between the study and control groups

Figure 1 depicts the distribution of critical thinking levels in the study and control groups before and during the intervention. Before the intervention, the majority of students in both groups displayed a high degree of critical thinking, with a higher proportion in the control group (86% vs. 72%). 28% of the study group and 14% of the control group indicated moderate critical thinking, whereas no students in either group were classified as having low critical thinking.

Following the intervention, there was a significant improvement in the study group, with the proportion of students with a high level of critical thinking increasing to 90% and the moderate level decreasing to 10%. In contrast, the control group showed a decrease in the proportion of students with a high level of critical thinking (70%) and an increase in the moderate level (30%), with no participants categorized as low in either group.

**Fig. 2:**
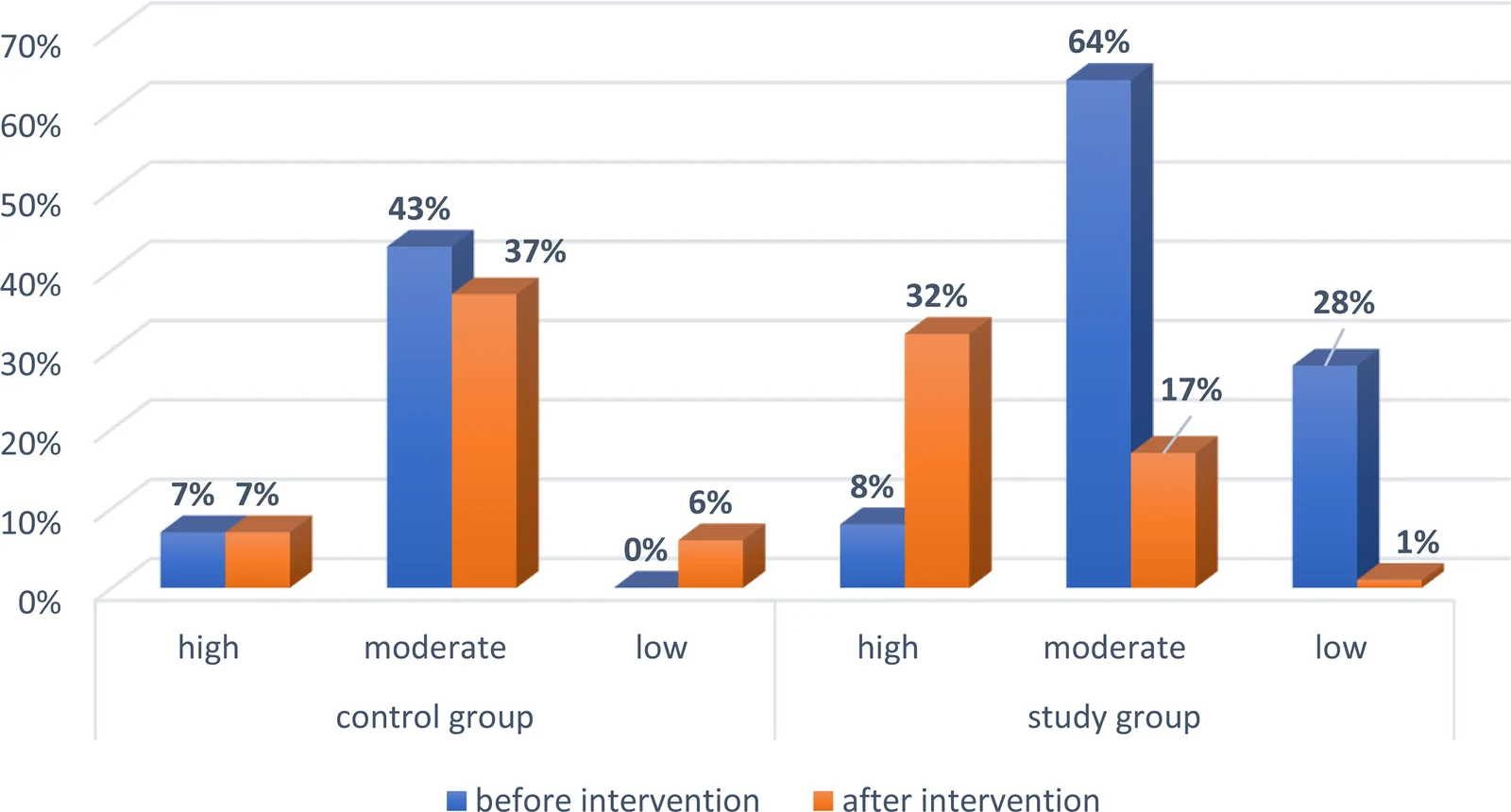
Levels of self-directed learning before and after intervention between the study and control group

The distribution of self-directed learning levels in the study and control groups before and during the intervention (Fig. 2). Before the intervention, the majority of students in the study group reported a moderate level of self-directed learning (64%), with only a tiny fraction reporting a high level (8%), and more than a quarter reporting a low level (28%). In the control group, the majority of pupils were rated as intermediate (43%), with no students classified as low.

After the intervention, the study group reported a significant increase in self-directed learning. The proportion of students with a high level climbed significantly to 32%, while the low level dropped to 1% and the intermediate level fell to 17%. In comparison, the control group showed very minor changes, with a tiny increase in the low level (6%) and a modest decrease in the moderate level (37%), while the number of students at the high level remained constant (7%).

## Discussion

The present study was guided by Vygotsky’s constructivist learning theory, particularly the concepts of social interaction, scaffolding, and the zone of proximal development. From this perspective, learning is not viewed as the passive reception of information but as an active, socially mediated process in which students construct meaning through guided inquiry, dialogue, reflection, and application. In the current intervention, the modified Know–Want–Learn–How–Apply–Questions (KWHLAQ) strategy served as a structured scaffold that supported students as they moved from what they already knew toward more complex levels of understanding, reasoning, and self-regulated learning. The K step activated prior knowledge, the W step helped students identify learning needs, the H step guided them to seek resources and learning strategies, the L step encouraged reflection on acquired knowledge, the A step supported application to clinical situations, and the Q step promoted further questioning and peer discussion. Thus, the instructor’s role was not merely to deliver content but to provide temporary structure, guidance, questioning, and feedback that could help students gradually assume greater responsibility for their own learning.

Within this theoretical framework, the present study found that the modified KWHLAQ strategy was associated with improved self-reported critical thinking among critical care nursing students. Before the intervention, there was no statistically significant difference between the study and control groups in total critical thinking scores, suggesting that both groups were relatively comparable at baseline. After implementation of the intervention, students in the study group reported significantly higher critical thinking scores than those in the control group. Moreover, the study group showed significant improvement in overall critical thinking after the intervention, whereas the control group showed a significant decline. These findings should be interpreted cautiously because critical thinking was measured using a self-reported instrument rather than through direct observation of students’ clinical reasoning, decision-making behavior, or performance in real clinical situations.

The improvement in self-reported critical thinking may be explained by the sequential and reflective nature of the KWHLAQ strategy. By moving students through stages of recalling prior knowledge, identifying learning gaps, searching for information, reflecting on what was learned, applying knowledge, and generating further questions, the strategy may have encouraged deeper engagement with learning content. In this way, KWHLAQ may have helped transform students from relatively passive receivers of information into more active learners who question, analyze, apply, and reflect on what they learn. However, because the study did not directly assess clinical performance or observed critical thinking behaviors, this explanation remains inferential and should be confirmed through future studies using objective measures such as performance-based assessments, simulation evaluation, clinical observation, or standardized critical thinking tasks.

The current findings also align with Bailey’s adaptation of the KWL chart in problem-based learning within nursing education. Bailey (2017) found that KWL provided nursing students with the needed structure when addressing the ambiguity of case studies and concluded that KWL has the potential to improve performance and critical thought in problem-based learning classrooms [27, 28]. This is particularly relevant to critical care nursing education, where students are expected not only to remember theoretical information but also to interpret patient data, prioritize problems, make judgments, and apply knowledge safely in rapidly changing clinical situations. Therefore, the critical care nursing context adds important educational relevance to the present study. Critical care content often requires students to integrate pathophysiology, assessment findings, urgent priorities, and evidence-based interventions. The modified KWHLAQ strategy may be especially suitable in this context because it offers a structured pathway for connecting theoretical knowledge with clinical reasoning and application.

The significant decrease observed in the control group may reflect the possible limitations of traditional teaching methods that depend more heavily on receiving information than on active inquiry, application, and reflection. Without a structured reflective tool, students may have fewer opportunities to connect prior knowledge with new information, identify gaps in understanding, or practice applying concepts to clinical situations. In contrast, the KWHLAQ strategy appears to provide a cognitive roadmap that helps students organize their thinking and monitor their learning. However, this interpretation should be treated with caution because the decline in the control group was also based on self-reported responses and may have been influenced by factors such as students’ motivation, test familiarity, academic workload, or perception of their own abilities.

The present study also showed that the modified KWHLAQ strategy was associated with improved self-reported self-directed learning among critical care nursing students. Before the intervention, the control group had significantly higher self-directed learning scores than the study group, indicating that the study group started from a relatively lower baseline. After the intervention, however, the study group showed a significant increase in total self-directed learning scores, whereas the control group showed no significant overall improvement. More importantly, after the intervention, the study group reported higher self-directed learning scores than the control group. These findings suggest that the modified KWHLAQ strategy may have supported students’ perceived ability to take responsibility for their own learning. However, because self-directed learning was measured through self-report, the results should not be interpreted as direct evidence that students actually changed their independent learning behaviors outside the classroom.

A key issue concerns the relationship between self-directed learning and the highly structured nature of the KWHLAQ strategy. At first glance, a structured teaching strategy may appear inconsistent with self-directed learning. However, self-directed learning does not necessarily mean unsupported or unguided learning, especially among undergraduate nursing students who are still developing learning autonomy. From Vygotsky’s perspective, external structure can function as scaffolding that helps learners gradually internalize planning, monitoring, questioning, and evaluation behaviors. In the present study, KWHLAQ provided an organized framework through which students practiced the essential components of self-directed learning. The strategy was externally structured at the beginning, but its purpose was to guide students toward greater internal regulation of their learning.

The improvement in self-directed learning may be related to the structure of the KWHLAQ strategy itself. The K step helped students recognize what they already knew, while the W step encouraged them to identify what they needed to learn. The added H step was particularly important because it required students to think about how they could learn more, search for information, and use available learning resources. The L step helped students evaluate what they had learned, while the A step encouraged them to apply knowledge in practical or clinical situations. Finally, the Q step promoted further inquiry by encouraging students to generate new questions.

The significant gains in all dimensions of self-directed learning suggest that the modified KWHLAQ strategy may have enhanced not only students’ knowledge acquisition but also their perceived learning behaviors. Improvement in motivation may have occurred because students became more actively involved in setting their own learning goals. Improvement in planning and implementation may be explained by the clear sequence of the strategy, which guided students to organize learning tasks and follow them systematically.

The increase in self-monitoring indicates that students perceived themselves as more able to evaluate their progress and recognize areas requiring further improvement. In addition, the improvement in interpersonal communication may be attributed to discussion, questioning, sharing ideas, and exchanging learning experiences during the intervention. In contrast, the control group showed inconsistent changes. Their planning and implementation scores significantly decreased, self-monitoring showed only minor improvement, and no significant changes were observed in learning motivation or interpersonal communication. These findings may reflect the limitations of traditional teaching strategies, which may not provide enough opportunities for students to plan their learning, monitor their progress, or actively interact with peers. However, these interpretations should be considered tentative because the study did not directly observe students’ independent learning practices.

Although the present study appears novel in examining the effect of the modified KWHLAQ strategy on self-directed learning among critical care nursing students, its findings are supported by previous studies using structured or learner-centered strategies in other educational contexts. Berger-Estilita et al. (2025) demonstrated that structured learning frameworks in health professions education enhanced interpersonal communication and collaborative engagement among learners, consistent with the gains observed in the present study’s interpersonal communication subscale [18]. The KWHLAQ strategy’s Q step, which explicitly prompts students to generate and share further questions, appears to operationalize this communicative dimension by creating structured opportunities for peer dialogue and idea exchange within the learning session.

Badakhshan et al. (2025) reported a significant positive association between self-directed learning ability and clinical competence among nursing students, reinforcing the clinical relevance of self-directed learning development as an educational outcome [14]. The present findings extend this evidence by suggesting that the KWHLAQ framework may serve as a structured educational vehicle for developing self-directed learning capacities, particularly planning, implementation, and self-monitoring, within the formal curriculum rather than leaving their development to incidental or informal learning experiences.

Finn et al. (2025), in a scoping review of self-directed learning in undergraduate clinical science programs, found that learner-centered strategies that explicitly scaffold goal-setting and self-monitoring behaviors were consistently associated with greater student engagement and academic motivation [10]. This aligns with the present findings, in which the study group demonstrated significant gains in learning motivation and self-monitoring following KWHLAQ implementation. These domains correspond directly to the W and L steps of the framework, as students identified learning needs and reflected on what they had learned.

### Limitations

Taken together, these limitations indicate that the findings should be interpreted with caution. Although the modified KWHLAQ strategy was associated with improvements in students’ self-reported critical thinking and self-directed learning, the quasi-experimental design, baseline differences between groups, summer-session context, reliance on self-report instruments, absence of follow-up assessment, and use of a single-institution convenience sample limit the strength and generalizability of the conclusions. Therefore, the present findings should be considered preliminary evidence suggesting that the modified KWHLAQ strategy may support students in becoming more active, reflective, organized, and responsible learners. Future studies using randomized or matched designs, larger and more diverse samples, objective performance-based assessments, and longitudinal follow-up are needed to confirm whether these perceived improvements translate into sustained behavioral changes and measurable gains in clinical reasoning, critical thinking, and self-directed learning.

### Implications for nursing education

The findings suggest that the modified KWHLAQ strategy may be integrated into nursing courses as a structured active-learning approach to support students’ self-reported critical thinking and self-directed learning. Nurse educators may use this strategy during lectures, clinical case discussions, problem-based learning sessions, simulation activities, and reflective assignments.

In relation to critical thinking, the KWHLAQ strategy may help students organize their thoughts, identify gaps in knowledge, ask relevant questions, analyze learning content, and consider how knowledge can be applied to clinical situations. These processes are particularly important in nursing education, especially in critical care courses where students are expected to interpret patient data, prioritize care, and make safe clinical judgments.

Nursing curricula may consider including structured active-learning strategies such as KWHLAQ to support student-centered education. The strategy may be used as a simple, low-cost teaching tool that can be adapted across theoretical and clinical nursing courses. It may also be incorporated into course activities, learning portfolios, clinical reflection sheets, and evaluation tasks to encourage continuous inquiry, reflection, organization of learning, and application of knowledge.

Further studies are recommended to examine the effectiveness of the modified KWHLAQ strategy with larger samples, different academic levels, and multiple nursing specialties. Future research should also compare KWHLAQ with other active-learning approaches, examine its long-term effect, and assess whether changes in self-reported critical thinking and self-directed learning are reflected in observable behaviors.

## Conclusion

The present study indicated that the modified KWHLAQ strategy was associated with significant improvements in nursing students’ self-reported critical thinking and self-directed learning. Regarding critical thinking, students who received the KWHLAQ intervention reported higher post-intervention critical thinking scores than those in the control group, suggesting that the strategy may support students’ ability to question, analyze, organize, reflect on, and apply learning content.

Therefore, the results suggest perceived improvement in critical thinking and self-directed learning, but they do not prove actual behavioral change. Future studies using objective and observational assessment methods are needed to determine whether these perceived improvements translate into measurable changes in students’ learning behaviors and clinical competence.

## Data Availability

The datasets generated and analyzed during the current study are available from the corresponding author on reasonable request.

## Abbreviations

CT: Critical thinking
CThQ: Critical Thinking Questionnaire
KWL: Know–Want–Learned
KWHLAQ: Know–Want–Learn–How–Apply–Questions
SDL: Self-directed learning
SDLI: Self-Directed Learning Instrument
ANCOVA: Analysis of covariance
ZPD: Zone of proximal development
